# Positional Mapping and Candidate Gene Analysis of the Mouse *Ccs3* Locus That Regulates Differential Susceptibility to Carcinogen-Induced Colorectal Cancer

**DOI:** 10.1371/journal.pone.0058733

**Published:** 2013-03-14

**Authors:** Charles Meunier, Lauren Van Der Kraak, Claire Turbide, Normand Groulx, Ingrid Labouba, Pablo Cingolani, Mathieu Blanchette, Garabet Yeretssian, Anne-Marie Mes-Masson, Maya Saleh, Nicole Beauchemin, Philippe Gros

**Affiliations:** 1 Department of Biochemistry, McGill University, Montreal, Quebec, Canada; 2 Goodman Cancer Research Centre, McGill University, Montreal, Quebec, Canada; 3 Centre de recherche du Centre Hospitalier de l'Université de Montréal et Institut du Cancer de Montréal, Université de Montréal, Montréal, Quebec, Canada; 4 McGill Centre for Bioinformatics, McGill University, Montreal, Quebec, Canada; 5 McGill Complex Traits Group, McGill University, Montreal, Quebec, Canada; Ohio State University Medical Center, United States of America

## Abstract

The *Ccs3* locus on mouse chromosome 3 regulates differential susceptibility of A/J (A, susceptible) and C57BL/6J (B6, resistant) mouse strains to chemically-induced colorectal cancer (CRC). Here, we report the high-resolution positional mapping of the gene underlying the *Ccs3* effect. Using phenotype/genotype correlation in a series of 33 AcB/BcA recombinant congenic mouse strains, as well as in groups of backcross populations bearing unique recombinant chromosomes for the interval, and in subcongenic strains, we have delineated the maximum size of the *Ccs3* physical interval to a ∼2.15 Mb segment. This interval contains 12 annotated transcripts. Sequencing of positional candidates in A and B6 identified many either low-priority coding changes or non-protein coding variants. We found a unique copy number variant (CNV) in intron 15 of the *Nfkb1* gene. The CNV consists of two copies of a 54 bp sequence immediately adjacent to the exon 15 splice site, while only one copy is found in CRC-susceptible A. The Nfkb1 protein (p105/p50) expression is much reduced in A tumors compared to normal A colonic epithelium as analyzed by immunohistochemistry. Studies in primary macrophages from A and B6 mice demonstrate a marked differential activation of the NfκB pathway by lipopolysaccharide (kinetics of stimulation and maximum levels of phosphorylated IκBα), with a more robust activation being associated with resistance to CRC. NfκB has been previously implicated in regulating homeostasis and inflammatory response in the intestinal mucosa. The interval contains another positional candidate *Slc39a8* that is differentially expressed in A vs B6 colons, and that has recently been associated in CRC tumor aggressiveness in humans.

## Introduction

The pathogenesis of colorectal cancer (CRC) is associated with the sequential accumulation of mutations in specific genes, which causes stepwise progression from pre-neoplastic lesions to full blown adenocarcinoma [1]. Histopathological stages correlating with somatic molecular rearrangements are well described [1], [2]. However, only in recent years and with the advent of genome-wide association studies has the degree of complexity in interactions between the genetic and environmental components contributing to the etiology of human colorectal cancer been appreciated [3], [4], [5], [6].

For a small proportion of CRC cases (<10%), a clear and highly penetrant genetic determinant can be observed in hereditary cancer syndromes, most importantly Familial adenomatous polyposis (FAP), Lynch syndrome (Hereditary non-polyposis colon cancer) and alternately, inflammatory bowel diseases (IBD)-linked CRCs [7], [8]. On the other hand, most CRC cases (>90%) are sporadic with no prior family history. The etiology of sporadic CRC involves two-way interactions between a complex genetic component, and poorly defined environmental factors [3], [6]. To date, as many as 16–20 common low-penetrance variants have been identified in genome-wide association studies (GWAS) for human sporadic CRC [9], [10]. Nearly half of those loci are tightly linked or allelic with components of the TGFß signaling pathway: SMAD7, GREM1, BMP2, BMP4, RHPN2 and LAMA5 ([11], [12], reviewed in [13]). On the other hand, it has been proposed that as many as 170 such loci may contribute to CRC susceptibility in humans [13].

Over 25% of all cancers are thought to be associated with chronic infection, inflammation or other types of inflammatory response [14]. Chronic inflammation has recently been appreciated as a major contributor to the etiology of CRC in humans [15], [16], reviewed in [13]. Thus, patients affected by inflammatory bowel diseases (IBD) have a much higher risk of developing colitis-associated (CA) CRC, the extent of the colitis manifestation correlating with the incidence of CA-CRC [17]. In addition, non-steroidal anti-inflammatory drugs (NSAID) show a protective effect against different types of cancers [18]. Interestingly, several key components of TGFβ-mediated Th17 and Th1 immune response pathways have recently been identified as low-penetrance loci associated with IBD onset, which could implicate TGFβ signaling in both IBD-linked as well as sporadic CRCs ([15], [16], reviewed in [19], [20]).

The mouse represents a valuable experimental model to dissect the complex genetic component of human CRC. Mice are available as inbred strains fixed for homozygosity for different allelic variants representing wide genetic diversity at key genes and pathways relevant to CRC pathogenesis. In addition, CRC can be induced in a reproducible and well-controlled fashion by chemical mutagens such as azoxymethane (AOM) [21], [22]. The resulting tumors closely resemble their human counterpart with respect to histopathology (from aberrant crypt foci to carcinoma *in situ*) and underlying genetic alterations (mutations in *Apc*, *Kras* and *ß-catenin*) [23], [24]. Inbred mouse strains show marked differences in susceptibility to carcinogen-induced CRC and classical linkage analyses in informative crosses have localized several loci that regulate inter-strain differences in susceptibility [25], for example, *Ccs1* [26], *Ssic1* [27], and *Ccs2* [28]. Parallel studies in congenic strains derived from BALB/cHeA and STS/A suggested a plurality of additional loci (*Scc1* to *Scc15*) affecting response to carcinogen-induced CRC [29], [30]. Of those, the positional cloning of the *Scc1* locus led to the identification of *Ptprj* as causative gene, and somatic rearrangements within the human homologue *PTPRJ* were identified in human CRC [31], [32].

In the AOM chemical carcinogenesis model, C57BL/6J strain (B6) is resistant with few CRC tumors noted 18 weeks following initiation of treatment (typically 0–5 tumors), while A/J (A) are highly susceptible with tumor multiplicity varying between 20–50 [33]. In our lab, we have used a set of AcB/BcA recombinant congenic mouse lines (RCS) derived from CRC-resistant B6 and CRC-susceptible A to identify the genetic determinants responsible for the differential susceptibility of these strains to AOM-induced CRC. The 13 AcB and 22 BcA strains were derived by systematic inbreeding from a double backcross (N3), and each strain contains a small amount (12.5%) of DNA from one parent fixed as a set of discrete congenic segments (mapped by genotyping) on the background (87.5%) of the other parent. Individual resistance/susceptibility loci contributing to a complex trait may segregate in individual RCS and can be studied in isolation, facilitating gene identification studies. This led to the mapping of three loci (*Ccs3*, *Ccs4*, *Ccs5*) regulating response to AOM-induced CRC in these strains [33], [34], [35]. The *Ccs3* locus determines initial susceptibility to AOM-induced CRC (appearance of adenomas), while the *Ccs5* locus modulates tumor multiplicity in animals bearing susceptibility alleles at *Ccs3* [34]. The *Ccs3* locus was mapped to a 14 Mb segment on the central portion of chromosome 3. This interval contains 94 annotated transcripts, and several of these genes show robust expression in the colon, itself regulated in a strain-specific fashion.

In the current study, we have conducted genetic analyses in AcB/BcA strains and in crosses derived from them to further narrow the size of the *Ccs3* genetic interval to 2.2 Mb. We have further characterized the genes in the interval by expression profiling and genomic DNA sequencing.

## Results

### Delineation of the Ccs3 interval in AcB/BcA recombinant congenic and AxB/BxA recombinant inbred strains

**Figure 1:**
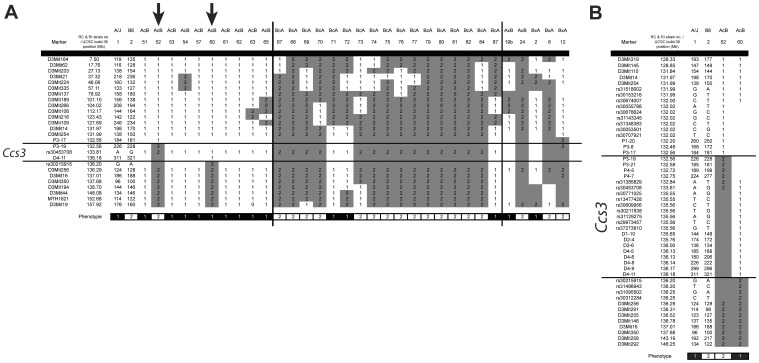
Haplotype structure at the *Ccs3* locus in recombinant congenic (RCS) and recombinant inbred (RI) strains. **A**. Chromosome 3 haplotypes of AcB/BcA strains are displayed along with their resistance (white) or susceptibility (black) status for AOM-induced CRC (bottom strip). The B6-derived chromosomal segments are indicated in grey (2), while the A haplotypes are annotated in white (1). Arrows (↓) indicate key RCS strains delineating the minimum chromosomal interval for the *Ccs3* locus. **B**. Detailed haplotype map of the *Ccs3* locus, including delineation of the proximal and distal boundaries by high-density SNP genotyping in key informative congenic strains (AcB52, AcB60).

Phenotyping a subset of 23 AcB/BcA strains for susceptibility to AOM-induced CRC initially showed that differential susceptibility of A and B6 mouse strains to CRC is regulated by a single locus designated *Ccs3*. In these studies, *Ccs3* was mapped to a 14 Mb segment on the central portion of chromosome 3 [33]. To better delineate the *Ccs3* genetic interval, we phenotyped additional AcB/BcA strains (bringing the total to 33 strains), as well as a subset of AXB/BXA strains (AXB19, AXB24, BXA2, BXA8, BXA12), with some of these strains bearing informative recombinant haplotypes in the *Ccs3* region. Groups of mice were treated with 1 weekly dose of AOM injection for 8 weeks, and 11 weeks later, animals were sacrificed, colons were collected and tumors were scored. Strains were stratified according to the number of tumors detected, as either low/intermediate (≤10 tumors) or high (>15 tumors) [33]. This bimodal strain distribution pattern was then superimposed onto the known haplotype combination of A and B6 alleles for distal chromosome 3 (*Ccs3*) in these strains. A summary of all available data from AcB/BcA and AXB/BXA strains is shown in Figure 1A. This analysis confirmed the critical role of *Ccs3* alleles in CRC susceptibility trait, and further identified strains AcB52 and AcB60 as carrying informative recombinant haplotypes further delineating the boundaries of the locus on the proximal and distal sides, respectively. To better delineate the recombination breakpoints in these strains, we developed several additional informative markers (microsatellite and SNP markers) in this region by genomic DNA sequencing of A and B6 parents (see Materials and Methods section). Using these markers, we further delineated the recombination breakpoints on the proximal side (AcB52), between markers P3-17 (pst. 132.558 Mb) and P3-19 (pst. 132.562 Mb), and on the distal side (AcB60) between markers D4-11 (pst. 136.18 Mb) and rs30215915 (pst. 136.20 Mb) (Fig. 1B). These studies further reduced the size of the maximal physical interval of the *Ccs3* locus to 3.64 Mb (P3-17 to rs30215915).

### High resolution positional mapping of the Ccs3 locus by progeny testing of informative backcross mice

**Figure 2:**
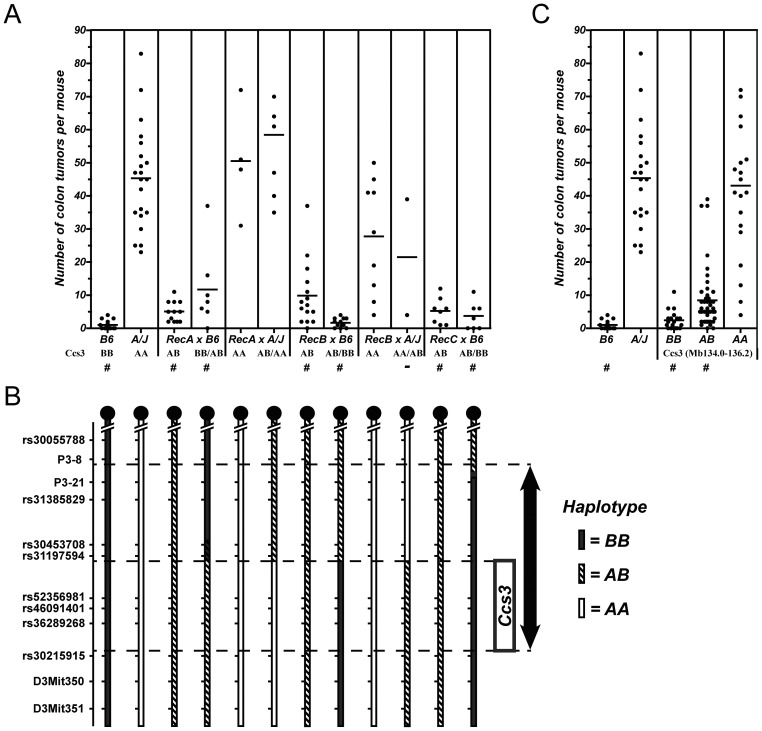
High resolution genetic mapping of the *Ccs3* locus in informative recombinant backcross mice. **A.** Following AOM treatment, colons were dissected, fixed and the number of tumors was scored (individual mice shown as ‘•’). Controls and backcross mice corresponding to key recombinants (Rec A, Rec B and Rec C) where bred to A/J (indicated A) and C57BL6/J (indicated as B). Mice were grouped by haplotype at the *Ccs3* locus and recombinant animals carry a different haplotype on each half of the *Ccs3* interval. Groups showing tumor numbers statistically different form the A parental group are identified (#). **B**. The *Ccs3* haplotype on distal chromosome 3 of each group of mice phenotyped in panel (A) is shown in black (B6), white (A/J) or striped (heterozygotes). Dashed lines show the interval first identified in RCS (arrow) as well as the reduced genetic interval for *Ccs3* suggested by a recombination event in Rec A and Rec B between markers rs31197594 and rs52356981, on the proximal side (box). The distal boundary was estimated from studies in recombinant congenic strains from Figure 1B. **C**. Aggregate genotype/phenotype correlation of pooled backcross mice for the reduced *Ccs3* interval (Mb134.0–136.2).

In these studies, we produced (B6xA)F2 animals, which were genotyped to identify informative recombinants within the *Ccs3* interval, using markers rs30055788 on the proximal side and rs30215915 on the distal side. Amongst a set of 240 F2 animals screened, we identified 3 informative recombinants which were designated RecA, RecB and RecC. Each recombinant was then backcrossed onto both B6 and A background, and multiple progeny from individual crosses were then genotyped for markers in the interval and phenotyped for susceptibility to CRC (Fig. 2A, 2B). In this analysis, the progeny of backcross between individual Rec mouse (A,B,C) and either A or B6 parents displayed a mixture of recombinant haplotypes in the region with combinations of homozygosity for A or B6 alleles or heterozygosity for A/B alleles (Fig. 2B). We then compared the genotype of the recombinant chromosomes with the phenotype of A and B6 backcrosses derived from them (Fig. 2A, 2B). Parental A controls developed high tumor numbers (X = 45.5; Fig. 2A) while B6 controls were low (X = 1.0; p<0.0001). Progeny testing of RecB and RecC backcrossed to B6 showed aggregate tumor numbers in these mice similar to B6 controls, in agreement with the homozygosity for B6 haplotypes in the distal portion of the previously defined *Ccs3* region. Conversely, progeny testing of RecA and RecB crosses to A showed tumor numbers in these animals that were not statistically distinct from those detected in parental A controls (although RecB X A were more intermediate), in agreement with homozygosity for the A allele on the distal portion of *Ccs3* (Mb134.0–136.2). Finally, we observed a third group of animals which displayed intermediate tumor multiplicity between that of the two parental extremes (X = 8.5; *p*<0.001 for either comparison); these included RecA x B6 (genotype BB proximal/AB distal; genotype AB proximal/AB distal), RecB X B6 (AB proximal, AB distal), RecB X A (AA proximal/AB distal), and RecC X A (AB proximal, AB distal). In this group of animals, there was a strong correlation between intermediate tumor multiplicity and heterozygosity for A/B haplotypes on the distal portion of the *Ccs3* interval, consistent with the co-dominant pattern of inheritance of *Ccs3* alleles we previously reported [33]. The combined effect of A/A, A/B and B/B alleles on the distal portion of *Ccs3* is shown in Figure 2C. These experiments further reduced the size of the *Ccs3* interval to ∼2.15 Mb, as delineated on the proximal side by reciprocal recombination events in RecA and RecB (in the rs31197594 and rs52356981 interval) and on the distal side the recombination event in AcB60 (in the rs31197594 to rs30215915 interval)(from Fig. 1).

### Positional candidates for the Ccs3 locus

**Figure 3:**
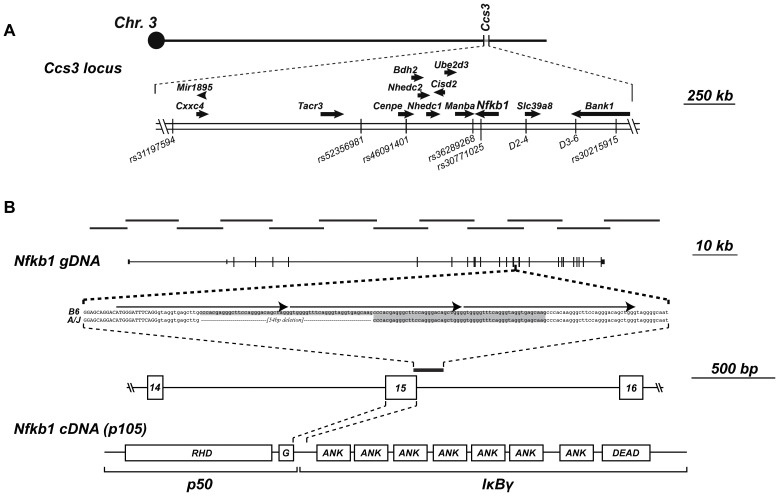
Positional candidate gene analysis of the *Ccs3* locus. **A**. The *Ccs3* locus contains 12 annotated transcripts, which position, direction of transcription (arrow), and position of reference SNP markers are shown. **B**. The position of long range amplification products used for deep sequencing of *Nfκb1* is shown to scale along with the position of the 25 coding and non-coding exons of *Nfκb1*. The position of the 54 bp intronic deletion identified in A/J is shown. It corresponds to the loss of a direct 54 bp repeat which is present in two copies in B6 (shaded in grey), which is itself composed of a 3-repeat structure (identified by arrows above the sequence). The position of the copy number variant with respect to exon 15 of the gene is shown, together with a projection of exon 15 translated sequence on predicted protein structure. Predicted p50 and IκBγ portions of the protein are shown, along with the Rel homology domain (RHD), ankyrin-repeats (ANK), death domain (DEAD) and glycine-rich region (G) separating p50 and p105 (called IκBγ).

The ∼2.15 Mb *Ccs3* interval contains 12 coding genes, one micro RNA (*Mir1895*), as well as several long non-coding RNAs (lincRNA) and one retroposon (Fig. 3A, and data not shown). The sequence of the 2.15 Mb segment was compared between A and B6 using reference genome sequences available from the Wellcome Trust Sanger Institute[36], and a complete list of all exonic, intronic and intergenic variants is presented in Table S1. There are no SNPs that distinguish A and B6 in either *Mir1895* (pst 133903469–133903547) nor in the lincRNAs and retroposon found at positions 134810372–134810477 (105 nt), 135099190–135100723 (1537 nt), 135158617–135158847 (231 nt), 135188928–135189496 (569 nt), 135390302–135391702 (1401 nt), 135626423–135626782 (360 nt) in the ENSEMBL datasets. Therefore, it is unlikely that these non-coding RNAs are responsible for the *Ccs3* effect, although a contribution of such non-coding RNAs cannot yet formally be excluded. Amongst the 12 annotated coding genes in the interval (*Cxxc4*, *Tacr3*, *Cenpe*, *Bdh2*, *Nhedc2*, *Nhedc1*, *Cisd2*, *Ube2d3*, *Manba*, *Nfkb1*, *Slc39a8*, *Bank1*), a number of nucleotide variants distinguish A and B6 (Table 1; Table S1), with single non-synonymous amino acid variants found in Manba (L844F) and Bank1 (A375M). Manosidase beta a (Manba) is a lysosomal enzyme, the inactivation of which causes beta-manosidosis, a lysosomal storage disease with a wide spectrum of neurological involvement[37]. Thus, a pathological variant in this gene is unlikely to cause susceptibility to CRC. On the other hand, the A375M variant in Bank1 (B cell scaffold protein with ankyrin repeats) is a conservative substitution that affects a residue non-conserved in Bank1 relatives (data not shown), and thus is unlikely to be pathological.

**Table 1:** List and position of polymorphisms prioritized for Ccs3 candidate genes.

Gene	Chr.	Position (mm9)	C57B/l6J	A/J	Polymorphism	aa sequence modification (conserved yes/no)	Known protein domain disrupted (confirmed yes/no)
Manba	3	135233296	C	T	Non-synonymous coding	L844F (yes)	No
Manba	3	135233708	G	T	3' UTR	-	
Manba	3	135233709	T	A	3' UTR	-	
Manba	3	135233980	T	G	3' UTR	-	
Manba	3	135233981	C	T	3' UTR	-	
Manba	3	135234039	G	A	3' UTR	-	
Manba	3	135234115	C	T	3' UTR	-	
Manba	3	135234181	C	CA	3' UTR	-	
Manba	3	135234235	T	C	3' UTR	-	
Manba	3	135234344	T	A	3' UTR	-	
Manba	3	135234360	G	A	3' UTR	-	
Nfkb1	3	135247860	C	CT	3' UTR	-	
Nfkb1	3	135253339	G	A	synonymous coding	-	
Nfkb1	3	135264465	A	G	synonymous coding	-	
Nfkb1	3	135266802	A	G	synonymous coding	-	
Nfkb1	3	135267953	GCCCACGAGGGCTTCCAGGGACAGCTAG GGTGGGGTTTCAGGGTAGGTGAGCAAG	G	Intronic	-	
Nfkb1	3	135271198	A	G	synonymous coding	-	
Nfkb1	3	135276630	T	C	synonymous coding	-	
Bank1	3	135876873	G	A	Non-synonymous coding	A375M (no)	No
Bank1	3	135876874	C	T	Non-synonymous coding	A375M (no)	No

**Figure 4:**
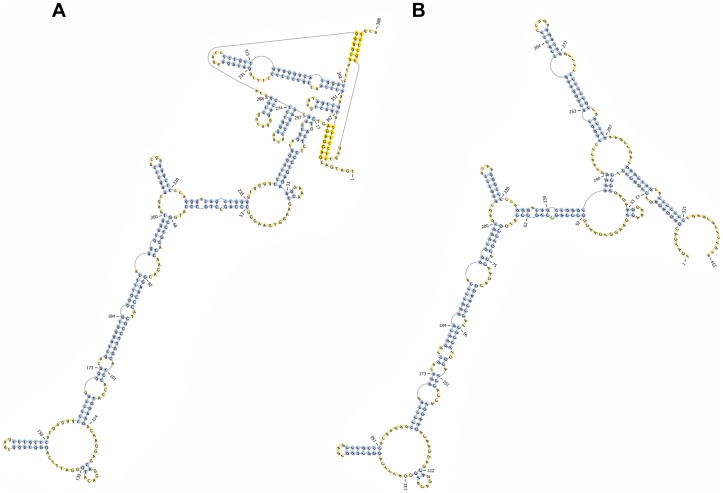
Secondary structure prediction of *Nfκb1* pre-mRNAs produced by AJ and B6 copy number variant. The pknotsRG tool [77] was used to generate secondary structures for the 350 nucleotides segment spanning the 3-repeat structure of B6 (**A**) and the deletion variant characteristic of A/J (**B**). Unpaired bases are indicated in blue, yellow bases identifying nucleotides involved in pseudoknot structures. This analysis identifies a pseudoknot with elaborate intra-molecular complementarity which is disrupted in the A/J variant allele.

**Figure 5:**
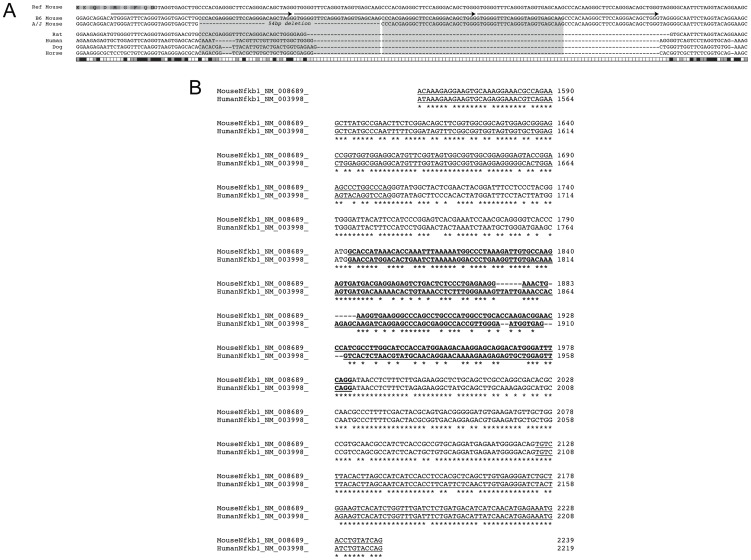
Cross-species sequence conservation of proximal intron 15 of the *Nfκb1* gene. **A**. ClustalW alignment (EBI) of *Nfκb1* intron 15 sequences from different species (shown at the left of the alignment). The reference mouse sequence from B6 is shown on top, including the single letter amino acid code for the polypeptide segment coded by exon 15. The genomic sequence of A is shown exactly as in Fig. 3B, including the deleted 54 bp duplication (‘---’) and the 3-repeat DNA motif overlapping the 3′ splice site of exon 15 (Arrows). Both mouse sequences (B6 and A) have been aligned to corresponding rat, dog, horse and human genomic sequences. Conservation of each mouse nucleotide is represented as shaded boxes at the bottom with high (black) to low (white) level conservation. **B**. *Nfκb1* cDNA sequence encoded by exon 13 to exon 17 (displayed as alternating underlined and regular format for each exon) of mouse and human genes have been aligned. The least conserved portion of the sequence is that encoded by exon 15, which sequence is in bold characters. Conserved nucleotides across the sequence are identified (*).

We have previously reported RNA transcript profiling studies, comparing expression of genes in the *Ccs3* interval both for A vs. B6 normal mucosa, and for normal mucosa vs. tumors from A mice[33]. Re-sequencing of all annotated coding exons and exon/intron boundaries was undertaken for genes displaying high expression in normal colonic mucosa (*Cisd2, Ube2d3*, *Nfκb1* and *Slc39a8*). Because of its prior association with colonic epithelium homeostasis, and inflammatory response *in situ*, the *Nfκb1* gene of our A mouse stock was sequenced in its entirety (130 kb). This combined analysis failed to identify nucleotide variants that affected consensus splice site sequences (Table S1), with the notable exception of a copy number variant (CNV) consisting of a 54 bp element located 13 nucleotides downstream the 3′ splice site of exon 15 (Fig. 3B). This element is present as two copies in B6 genomic DNA, but one copy is missing from the corresponding position in A. The duplicated 54 bp element found in B6 is itself part of a repetitive DNA motif composed of 3 close-to-identical DNA repeats that includes the 3′ splice site of *Nfκb1* exon 15 in the B6 genome (Fig. 3B). The deletion of one of the two 54 bp elements in A disrupts the integrity of the 3-repeat motif found in B6 DNA. The variability in the number of those close-to-identical DNA repeats suggested a possible shift in secondary sequence conformation at this junction of exon 15/intron 15 in genomic DNA and/or precursor RNA. Indeed, preliminary analysis of secondary structure of a DNA fragment over 350 bp spanning the 3-repeat motif shows the presence of a putative pseudoknot in B6 mice (Fig. 4A) which is absent from the A genomic DNA (Fig. 4B). Furthermore, the 54 bp-element, one copy of which is absent in A, displays cross-species sequence similarity between mice, human and several other species (Fig. 5A), suggesting a possible conserved role of this element and associated secondary structure across several species. Interestingly, there appears to be significant sequence conservation of intron 15 across species, while the nucleotide sequence and predicted *Nfκb1* exon 15 amino acid sequence shows poor cross-species conservation (Fig. 5A, 5B). The specific role by which this CNV would regulate Nfkb1 function was investigated but, so far, no clear mechanism has been identified (see Discussion).

### Activation of the Nfκb Pathway

**Figure 6:**
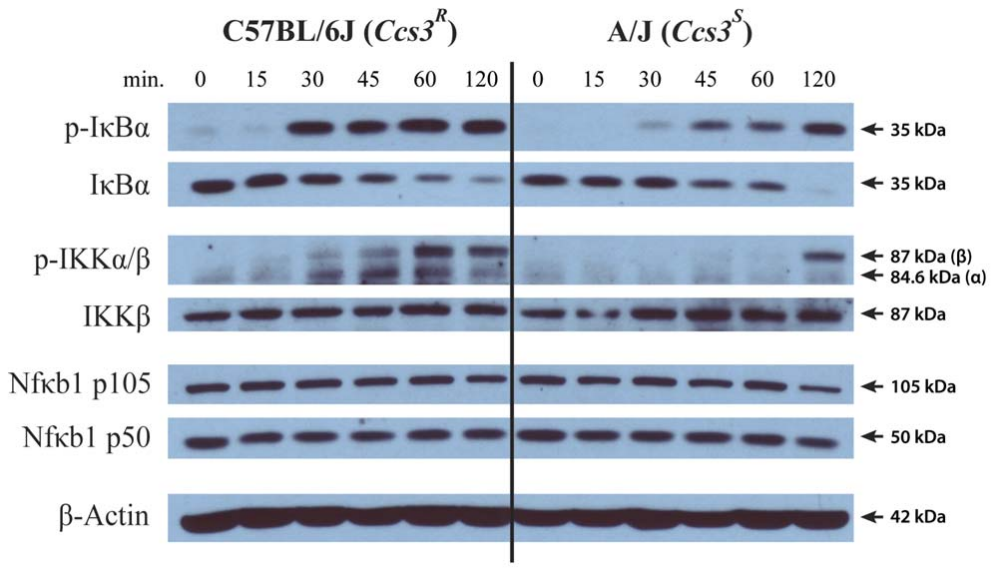
Activation of the Nfκb pathway in primary cells from A/J and C57BL/6J mice. Bone marrow-derived macrophages were prepared from A (*Ccs3^S^*) and B6 (*Ccs3^R^*) mice and were stimulated in the presence of lipopolysaccharide (LPS). At the indicated time intervals (minutes, shown at the top), cells were harvested, lysed and total protein extracts were prepared. Samples were resolved on 10% SDS-PAGE and analyzed by immunoblotting with antibodies against NFκB (p50, p105), total and phospohorylated (p-)IκBα as well as total and phosphorylated IκB kinases, (p-)IKKα/β and β-actin as loading control. The molecular weight of individual proteins is shown on the right of each assembled panel, each of which is representative of 3 independent experiments.

We also investigated activation of the Nfκb pathway in A and B6 strains, using a standard LPS induction assay in primary macrophages (BMDM). Induction of Nfκb in macrophages and in intestinal epithelial cells is very similar with respect to induction signals that are active in both cells (LPS/TLR4; NOD1/Peptidoglycan; TNFα/TNFα-R) and time kinetics[38]. BMDM were exposed to LPS, and at different time points, cells were lysed and analyzed by western blot for the expression of p50 and p105 Nfκb1 isoforms, total and phospohorylated (p-)Iκbα as well as the total and phosphorylated Iκb kinase, (p-)Iκkβ (Fig. 6). These experiments showed similar levels of expression of Nfκb1 p50 and p105 proteins, and total Iκkβ. These levels remained similar throughout the duration of the treatment. However, activation of the Nfκb pathway by LPS was much stronger in B6 than in A macrophages, as determined by the appearance of activated p-Iκkβ and the simultaneous decrease in Iκbα along the detection of p-Iκbα, targeted for degradation. In B6 macrophages, Nfκb activation occurred more rapidly and was more robust than in A BMDM (kinetics of appearance and total amount of p-Iκbα and p-Iκkβ). Overall, these results identify weaker Nfκb activation for A cells compared to B6 cells in response to stimulation by microbial LPS.

### Expression of Nfκb1 p105/p50 proteins in normal mucosa and in tumors from A/J

**Figure 7:**
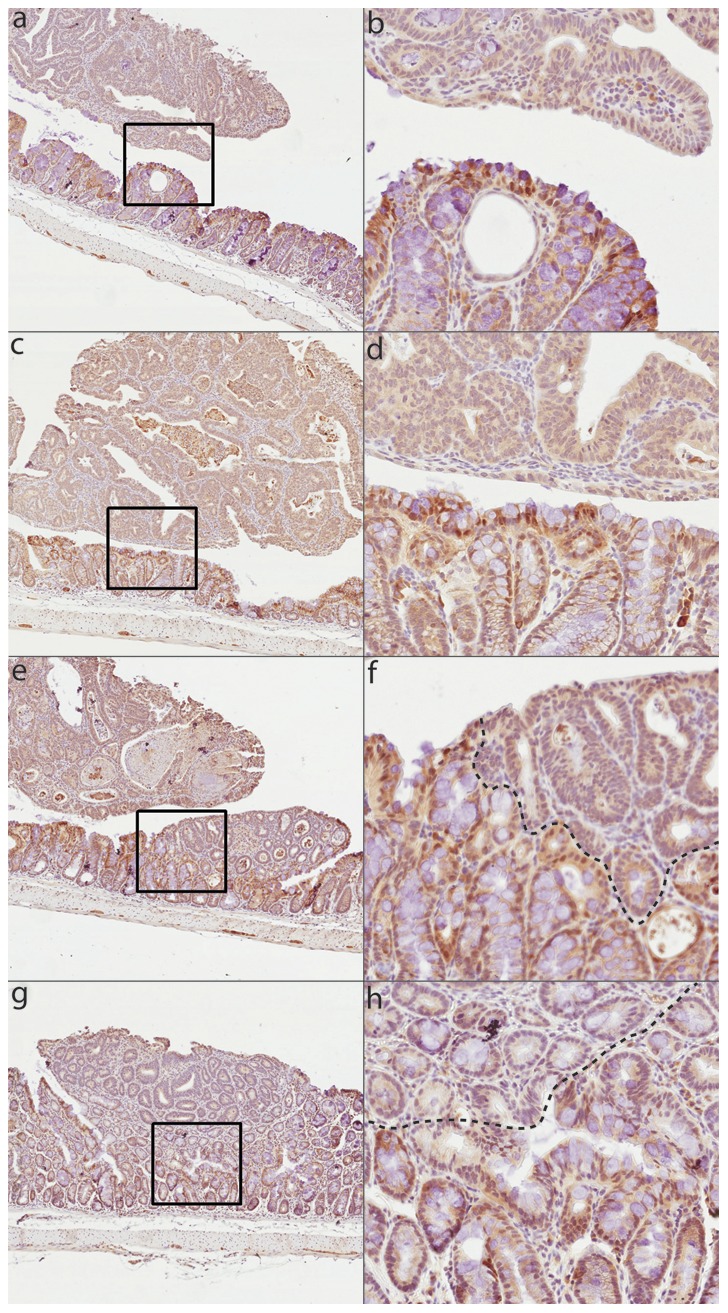
Nfkb1 protein expression in tumors and adjacent normal mucosa. Representative immunohistochemical staining (IHC) for p105/p50 Nfkb1 protein in tumors and adjacent colonic epithelium from mice carrying homozygous A/J-haplotype at Ccs3. 5× magnification (a, c, e, g) and corresponding 20× magnification (b, d, f, h) are shown for five representative tumors.

We investigated expression of the Nfκb1 p105 precursor by immunohistochemistry, using an antibody (see Materials and Methods) that recognizes both p105 and the p50 Nfκb1 product. In this analysis, we included on the same sections both normal mucosa, dysplastic lesions and more advanced adenocarcinomas either intramucosal or protubing in the intestinal lumen, all obtained at necropsy from A mice 18 weeks post-treatment. Several representative images are shown in Figure 7. In normal mucosa, Nfκb protein staining is seen in crypts, predominantly in nuclei of intestinal epithelial cells (IEC), and can also be detected in sub-population of lamina propria cells. This staining is largely absent in adjacent areas of tissue hyperplasia/adenomas (Fig. 7f/h), as well as in sections with further developped tumors (adenocarcinomas) seen in the intestinal lumen (Fig 7b/d). These results indicate a loss of Nfκb1 protein expression in cancerous lesions with low and high-grade dysplasia observed in A/J mice.

## Discussion

In recent years, genome-wide association studies (GWAS) have pointed at an impressive plurality of genetic factors contributing to CRC susceptibility in humans [4], [5]. In addition, it is increasingly recognized that environmental factors, such as diet, life-style and microbial flora can further contribute to modulate penetrance or expressivity of genetic pre-disposition. The contribution of individual genes to CRC susceptibility can be assessed in relevant mouse models, where both genetic and environmental factors can be controlled [6]. Likewise, the ‘forward genetic’ dissection of differential susceptibility of inbred strains to CRC may identify novel gene effects, the relevance of which can be subsequently tested for human CRC [21].

In the current study, we have reduced the size of *Ccs3* to ∼2.15 Mb, a size amenable to positional cloning [39]. This interval contains 12 annotated transcripts, of which 6 are expressed in the intestine (Fig. 3A). One of them encodes p105 Nfκb1, a strong positional candidate and central component of Nfκb signaling. Although many polymorphic variants were identified between A and B6 for genes in the interval, no obviously pathological missense or nonsense variants were identified in their coding regions. Immunohistochemical staining (IHC) revealed strong nuclear Nfκb1 expression in the normal colonic epithelium, whereas adjacent tumors in the same tissue expressed very low level Nfkb1 protein (Fig. 7). This suggests that colon tumorigenesis is associated with down-regulation of p105 Nfκb1 in our mouse model of AOM-induced CRC. In addition, we have detected a deletion near *Nfκb1* exon 15 in A mice. The 54 bp intronic deletion maps within a 3-unit repeat structure that overlaps the 3′ splice site of *Nfκb1* exon 15. The corresponding sequence of intron 15 shows notable cross-species conservation, and the segment affected by the deletion is predicted to form a very stable secondary structure that is disrupted in the A genome. DNA elements present in introns are known to influence RNA processing as sequence elements [40] or as secondary structures which can be bound by dsRNA binding proteins [41], [42]. Additionally, secondary DNA structure such as ssDNA hairpin loops can also affect DNA-protein recognition [43], gene expression and DNA recombination [44]. Hairpins formed in DNA repeats have been associated with a number of genetic disorders [45], [46], [47]. Importantly, protein expression studies in LPS-treated primary macrophages from A and B6 mice demonstrate differential activation of the Nfκb pathway in these cells. When monitoring time-dependent activation and maximal accumulation of activated p-Iκkβ and p-Iκbα targeted for degradation we noted that a more robust activation of the Nfκb pathway is associated with a marked decrease in susceptibility to CRC. Therefore, the convergence of genetic mapping data placing *Nfκb1* within the ∼2.15 Mb interval of *Ccs3*, the known role of the Nfκb pathway in homeostasis of the intestinal mucosa (see below), the detected loss of Nfκb p105/p50 protein expression in tumors compared to normal mucosa, the observed differential activation of this pathway in animals of different *Ccs3* haplotypes, and the presence of a distinguishing genetic alteration in the gene, together point at the *Nfκb1* as a very strong candidate for the *Ccs3* effect.

Nfκb1 is a member of the Nfκb family of transcription factors that share an amino-terminal DNA binding and dimerization Rel homology domain. However, it lacks a transcription activation domain (TAD) and relies on heterodimerization with TAD-containing Nfκb family members (p65/RelA, RelB, c-Rel) to activate transcription [48]. Nfκb1 is synthesized as a p105 precursor. Prior to activation, Nfκb1 dimers (p105) are associated with Iκb proteins: Iκbα, Iκbβ, Iκbε, and the precursor protein p100 (Nfκb2). These partners maintain the Nfκb1 dimers in the cytoplasm. Upon stimulation by TNFα, IL-1 and LPS, the IκB kinase (Iκk), assembled from 3 subunits (Iκkα, Iκkβ, Nemo), phophorylates Iκb proteins, including the precursor p105, which is then ubiquitinated and targeted for proteosomal degradation. This results either in complete degradation of p105 or its shortening into the p50 transcription factor (reviewed in [49]). This allows the nuclear translocation of p50, p65 and c-Rel containing dimers by nuclear translocation signal and induction of the expression of Nfκb target genes. The Nfκb1 p50 transcription factor is produced by limited proteolytic processing of the p105 precursor [50]. The p50/p105 ratio within the cell is important for function, since p105 acts as a Iκb protein, whereas p50 is essential to form transcription-activating Nfκb heterodimers; excess amounts of p50 (though devoid of a TAD) can have both inhibitory as well as stimulatory effects [49], [51].

In humans and mice, NFκB is a key signalling molecule regulating several aspects of the intestinal epithelium, including homeostasis, inflammatory response, and more recently development of neoplastic lesions in response to different stimuli [52]. The role of the NFκB pathway in regulating inflammatory response is complex, dosage- and context-dependent, acting both as a positive and negative regulator of inflammation. The pro-inflammatory function of NFκB is well established: a) the pathway is induced by microbial products and pro-inflammatory cytokines, b) NFκB binding sites are found in the regulatory regions of genes coding for cytokines and chemokines, and members of other pro-inflammatory pathways, c) NFκB proteins and pathway are constitutively activated in several chronic inflammatory conditions, and members of the NFκB pathways have been detected as pre-disposing alleles in human GWAS studies of such conditions including inflammatory bowel diseases, rheumatoid arthritis, psoriasis and others, d) inhibition of the NFκB pathway generally ameliorates inflammatory conditions (reviewed by [53]). In particular, a 4 bp deletion in the promoter region of human *NFκB1*, correlated with disrupted protein binding and decreased promoter activity *in vitro*, was associated to increased susceptibility to develop ulcerative colitis (UC) comparing UC-affected patients to healthy controls (Odds ratio of 1.59 for two different patient cohorts) [54]. On the other hand, inactivation of the NFκB pathway has also been associated with increased inflammation: a) mice lacking Iκkβ expression in intestinal epithelial cells (IEC) show increased susceptibility to chemical-induced colitis[55]; b) mice lacking Iκkγ/Nemo, and without Nfκb activity in IEC develop spontaneous colitis[56]; c) ablation of Iκkγ in keratinocytes is associated with psoriasis in mice[57]; d) Iκkβ deletion causes progressive neutrophilia, with increased IL1β expression and loss of inflammasome down-regulation in Nfκb-deficient myeloid cells[58], [59], [60].

Likewise, NFκB has been shown to have both a pro- and anti-tumorigenic role in malignant cells. For example, the *v-Rel* viral oncogene is the homolog of c-Rel, one of the NFκB subunits[61]; also, mutations in NFκB subunits themselves or in components that activate NFκB are associated with a variety of hematological malignancies[62], [63], [64]; activating mutations in upstream regulators such as CARD11 (inflammasome component) or MYD88 (constitutive TLR signaling) are associated with B-cell lymphoma, and downstream targets of NFκB are often mutated in multiple myeloma as well [61], [65], [66], [67]. Finally, in a mouse model of colitis-associated CRC (AOM plus dextran sulfate), loss of Iκkβ in IEC is linked to decreased tumor incidence, while loss of Iκkβ in macrophages leads to decreased tumor multiplicity and tumor size [55]. On the other hand, ablation of Iκkβ in IEC has no effect on cell proliferation *per se* [55]. Also, inhibition of Nfκb in hepatocytes in a diethyl nitrosamine chemical model enhances cyclin D1 expression and cell proliferation[68], while blockade of Nfκb through overexpression of Iκbα promotes Ras-induced epidermal growth resembling squamous cell carcinoma[69]. Also, hepatocyte-specific ablation ot Iκkγ results in spontaneous hepatocellular carcinoma[70], [71]. Such examples of anti-tumorigenic activity of Nfκb support *Nfκb1* as a positional candidate for *Ccs3*, including the reduced activation of this pathway in response to bacterial endotoxin detected in primary cells from A vs. B6 mice.

The mechanism by which the detected CNV in intron 15 of *Nfκb1* would be associated with differential activation and function of the Nfκb pathway in A vs. B6 primary cells remains unknown and awaits further study. However, we have observed that the A and B6 alleles at this CNV do not have a detectable effect on a) overall expression of *Nfκb1* RNA in normal mucosa (microarray data)[33], b) level of p105 or p50 proteins expressed by primary macrophages either constitutively or in response to LPS (Fig. 6), c) splicing of exons 14–16, as determined by exon chip analysis[33], and following re-construction of the two variants into appropriate expression constructs, transient transfection in HeLa cells, and analysis of spliced products by RT-PCR (data not shown). We have not yet tested the effect of the CNV alleles on other aspects of mRNA biology, including nuclear export and/or translatability in primary IEC.

While the sum of the evidence supporting *Nfkb1* as the dominant positional candidate for *Ccs3* is very strong, the reduced *Ccs3* interval delineated in our study retains another interesting positional candidate, *Slc39a8*. Slc39a8 is a member of a member of the Slc39 family of metal transporters that acts as an import system for Mn^2+^, Cd^2+^, Zn^2+^, and other divalent transition metals. *Slc39a8* RNA is broadly expressed in different tissues and cells, and in transfected cells, the protein is present at the apical pole where it functions as a (HCO_3_^−^)_2_ -dependent metal symporter[72]. Following expression profiling of all positional candidates by Affymetrix, *Slc39a8* remains the only transcript in the *Ccs3* interval that is differentially expressed in A (low) vs. B6 (high), but also in normal mucosa (high) vs tumors (low) derived from A [33]. In a recent genomic study of 276 human colorectal cancers by whole exome sequencing, DNA copy number, promotor methylation and mRNA expression, *SLC39A8* expression was found to be associated with decreased tumor aggressiveness score, as expressed by tumor stage, lymph node status, lymphatic and vascular invasion, and histology[73]. Genome-wide, *SLC39A8* (p<4.3×10^−10^) was one of only 19 genes that passed a combined highly significant statistical association (p<10^−9^) with tumor aggressiveness [73]. A direct role of *Slc39a8* in CRC and as a candidate for *Ccs3* will need to be assessed in mutant mice that bear mutant alleles at the *Slc39a8* locus, recently made possible with the creation of viable hypomorph allele [74].

Finally, we cannot formally exclude the possibility that the *Ccs3* effect may be caused by a combination of independent contributions from two or more closely linked genes within the interval identified in this study, including but not limited to *Nfkb1* and/or *Slc39a8*. Additional experiments will be required to formally identify the gene or combination of genes responsible for the *Ccs3* effect. In particular, the creation of sets of transgenic mice carrying overlapping cloned genomic DNA segments from the *Ccs3* region, and which transfer causes appearance of a CRC susceptibility phenotype of the donor strain, will constitute the final proof for the identity of the gene.

## Materials and Methods

### Ethics Statement

All animals were maintained at the Animal Care Facility of McGill University according to the guidelines of the Canadian Council on Animal Care (CCAC) and the animal protocol for this study was approved by the McGill University Animal Care Committee (UACC, protocol no. 5183).

### Mice

Inbred A/J (A), C57BL/6J (B6) and (B6×A/J)F1 mice were purchased from the Jackson Laboratory (Bar Harbor, ME, USA). The AcB/BcA set of RCS were derived from a double backcross (N3) between A and B6 parents at McGill University. The breeding, genetic characteristics and genotype of these animals for 625 markers have been previously described [75]. (B6×A/J)F2 mice were generated by brother-sister matings from a (B6×A/J)F1 hybrid. They were fed regular rodent chow (Charles River, St. Louis, MO) and water ad libitum.

### Carcinogen treatment and colon tumor preparations

As described previously [33], mice were treated with one weekly intraperitoneal (i.p.) injection of the carcinogen AOM (Sigma, St Louis, MO, USA) at 10 mg/kg for 8 weeks. Animal status and weights were monitored regularly each week. Animals were sacrificed 19 weeks following the first injection, colons were collected and opened longitudinally such that representative pictures could be captured and fresh material collected. Subsequently, the entire colon was fixed in 10% phosphate-buffered formalin and scored for the number of tumors and hyperplastic lesions.

### Preparation of tissues and immunohistochemical (IHC) staining

Mice were euthanized and intestines were immediately removed, washed in PBS and fixed in 10% phosphate-buffered formalin and processed for histology and for immunohistochemistry analysis. Fixed tissues were dehydrated in ethanol and embedded with paraffin, and 4μm sections were prepared. Tissue slides were incubated with primary NFκB p50 (NLS) antibody (1∶50 dilution of sc-114; Santa Cruz Biothechnology, Santa Cruz, CA, USA)), diluted 1∶50 in 1× PBS, for 90 min at 20°C in a humid chamber. Tissue slides were then incubated 20 min with secondary biotinylated antibodies (Dako Cytomation Inc). An additional incubation of 20 min with streptavidin-HRP reagent allowed revealing immunochemical staining by adding diaminobenzidine chromogen reagent (Dako Cytomation Inc). Diluted Harris haematoxylin (1∶2 in distilled water) was used for counterstaining the nuclei. Stained samples were dehydrated in ascending gradient of ethanol and toluene. High-resolution digital images of each tissue slide were generated using a whole-slide scanner (SanScope XT automated high-throughput scanning system from Aperio, CA, USA).

### Linkage analysis in AcB/BcA recombinant congenic strains

The published genetic map of the AcB/BcA RCS set [75] was used to identify the *Ccs3* susceptibility locus associated with AOM-induced CRC (described in [33]). The markers with highest linkage values were subsequently positioned on the February 2006 mouse (*Mus musculus*) genome data obtained from the Build 36 assembly by NCBI to attribute physical genomic positions. Several polymorphic SNP markers were also selected from the Mouse Phenome Database (phenome.jax.org) and genotyped to complement the haplotype map of distal chromosome 3. Fine-mapping of the locus interval was appraised by visual tracking of haplotypes in contributing and non-contributing strains.

### Genotyping of polymorphic markers designed in house

Genomic DNA was prepared by standard proteinase K protocol [75]. Microsatellite markers were genotyped by standard PCR-based methods using (α-^32^P) dATP labeling and separation on denaturing 6% polyacrylamide gels [75]. SNP markers were genotyped by PCR amplification and automated DNA sequencing (McGill University and Genome Quebec Innovation Centre, Montreal, QC). Fine-mapping analysis of the recombination events on the *Ccs3* proximal and distal end was performed using markers designed in house based on the UCSC Genome Bioinformatics DNA sequence (genome.ucsc.edu). Dinucleotide markers were identified as proximal (P) or distal (D) along with their position relative to one another (Px-xx, Dx-xx). Fine mapping of the recombination event in Rec A and Rec B mice was examined using additional SNP markers obtained from Mouse Genome Resequencing Project (Wellcome Trust Sanger Institute; sanger.ac.uk).

### Sequencing of coding exons of Ccs3 candidate genes

For each of the positional candidates, sequence-specific oligonucleotide markers covering individual exons and splice sites sequences were designed (primer3.sourceforge.net). Sequences were amplified by conventional PCR with tail DNA from A and B6 inbred mice, and the sequence of the PCR products was determined by automated DNA sequencing (McGill University and Genome Quebec Innovation Centre, Montreal, QC).

### Genomic DNA sequencing (exonic and intronic sequences) of Nfκb1 candidate gene

Primers for long-range PCR were obtained from Perlegen genome resequencing database (mouse.perlegen.com) (See Table S2). A region measuring 130 Kb covering *Nfκb1* gDNA and 10 kb flanking sequences was amplified as 12 overlapping fragments of 10–12 kb (Fig. 4B) from A DNA using Fermentas Long PCR Enzyme Mix (#K0182; Fermentas, Burlington, ON, Canada). Once amplified, libraries were prepared using Roche Rapid Library kits (Mississauga, ON, Canada) according to the manufacturer's specifications. Briefly, PCR products were fragmented by nebulization to about 600 bp on average. After end-polishing, adapters were ligated (using MID11) and the resulting library was used as a template for emulsion PCR. The sequencing reaction was performed at the McGill University and Genome Quebec Innovation Centre and consisted of 200 cycles with the Titanium chemistry (Roche GS-FLX Titanium).

Over 87 000 reads with an average of 368 bases (32.2 Mb total) were obtained on 1/8 region of a plate. Basic quality control analysis was performed using in-house tools. Reads were mapped to the reference genome mm37.59 (from ENSEMBL). A total of 73 016 reads where mapped to the reference sequence (83:4% of the original reads). Using these mapped reads, we created a list of putative SNPs (a.k.a. pileup). SNPs with quality lower than 200 were filtered out (arbitrary threshold). After filtering out low quality predictions, the effect of the remaining 236 SNPs was predicted using SnpE (snpeff.sourceforge.net). Coverage variations analysis was performed in order to find long deletions (over 10 bases). We used a sliding window average to enumerate regions with coverage lower than a threshold, which allow us to detect the presence of a single indel, a 54 bp-deletion downstream of exon 15 of *Nfκb1*. Any sequence displaying lower density coverage were repeated with amplification by standard PCR (Primers listed in Table S2) followed by Sanger sequencing.

### Macrophages, LPS treatment and analysis of NFκB activation

Bone marrow derived macrophages (BMDM) were prepared from femurs of 12- to 16-weeks-old B6 and A mice in bacteriological-grade Petri dishes (Fisher) with 20% L-cell-conditioned medium (LCCM) according to standard protocols [76]. After 7 days, cells were harvested by gentle washing of the monolayer with PBS-citrate and plated in 6-well cell culture dishes (2.5×10^6^ cells/wells; Corning) to be cultured for an additional 16–24 hours. BMDM were exposed to lipopolysaccharide (LPS, 100 ng/mL; Sigma L-2630) in Opti-MEM medium (Gibco) for a time course of 120 min., harvested and lysed directly in 2× Laemmli sample buffer. After electrophoretic separation and transfer, proteins were detected with either phospho-specific antibodies for pIκBα (9246 s) and pIKK-α/β (2697) in 2% BSA-containing TBST (10 mM Tris–HCl pH 8, 150 mM NaCl, 0.1% Tween 20) or with antibodies against IκBα (4812), IKK-β (2370), β-actin (A1978; all from Cell Signaling Technologies) and NFκB p50/p105 (sc-114; Santa Cruz). Protein expression was visualized by enhanced chemiluminescence (Super-Signal West Pico kit, Thermo Scientific).

### Statistical analysis

Differences in tumor number between groups of mice was evaluated for statistical significance by Mann-Whitney t-test. Results were considered statistically different for p≤0.05.

## Supporting Information

Table S1 **List and position of all polymorphisms distinguishing CRC-susceptible A/J from CRC-resistant C57Bl/6J in the Ccs3 interval.** Type of polymorphisms are indicated with individual allele for each strain using Sanger [36] and in house datasets. For large structural variant calls (SV, >100 bp), beginning and end of sequence is indicated at each respective position (i.e. 2 entries per polymorphic change) where the average middle position is indicated in B6 and the polymorphic change is given for A/J. Additional abbreviations were used for single nucleotide polymorphisms (SNP), short indels (indel, <100 bp) and transposable element (TE).
(XLSX)

Table S2 **List of primers used for sequencing Nfkb1 130 kb genomic region.**
(XLS)
